# Deep Learning-Based Versus Iterative Image Reconstruction for Unenhanced Brain CT: A Quantitative Comparison of Image Quality

**DOI:** 10.3390/tomography9050130

**Published:** 2023-08-31

**Authors:** Andrea Cozzi, Maurizio Cè, Giuseppe De Padova, Dario Libri, Nazarena Caldarelli, Fabio Zucconi, Giancarlo Oliva, Michaela Cellina

**Affiliations:** 1 Service of Radiology, Imaging Institute of Southern Switzerland (IIMSI), Ente Ospedaliero Cantonale (EOC), Via Tesserete 46, 6900 Lugano, Switzerland; andrea.cozzi@gmail.com; 2 Postgraduation School in Radiodiagnostics, Università degli Studi di Milano, Via Festa del Perdono 7, 20122 Milano, Italygiuseppe.depadova@unimi.it (G.D.P.); dario.libri@unimi.it (D.L.); nazarena.caldarelli1@unimi.it (N.C.); 3 Department of Radioprotection, Fatebenefratelli Hospital, ASST Fatebenefratelli Sacco, Milano, Piazza Principessa Clotilde 3, 20121 Milano, Italy; fabio.zucconi@asst-fbf-sacco.it; 4 Radiology Department, Fatebenefratelli Hospital, ASST Fatebenefratelli Sacco, Milano, Piazza Principessa Clotilde 3, 20121 Milano, Italy; linforisonanza@gmail.com

**Keywords:** brain computed tomography, iterative reconstruction algorithms, deep learning-based reconstruction algorithms, image quality, image noise

## Abstract

This exploratory retrospective study aims to quantitatively compare the image quality of unenhanced brain computed tomography (CT) reconstructed with an iterative (AIDR-3D) and a deep learning-based (AiCE) reconstruction algorithm. After a preliminary phantom study, AIDR-3D and AiCE reconstructions (0.5 mm thickness) of 100 consecutive brain CTs acquired in the emergency setting on the same 320-detector row CT scanner were retrospectively analyzed, calculating image noise reduction attributable to the AiCE algorithm, artifact indexes in the posterior cranial fossa, and contrast-to-noise ratios (CNRs) at the cortical and thalamic levels. In the phantom study, the spatial resolution of the two datasets proved to be comparable; conversely, AIDR-3D reconstructions showed a broader noise pattern. In the human study, median image noise was lower with AiCE compared to AIDR-3D (4.7 vs. 5.3, *p* < 0.001, median 19.6% noise reduction), whereas AIDR-3D yielded a lower artifact index than AiCE (7.5 vs. 8.4, *p* < 0.001). AiCE also showed higher median CNRs at the cortical (2.5 vs. 1.8, *p* < 0.001) and thalamic levels (2.8 vs. 1.7, *p* < 0.001). These results highlight how image quality improvements granted by deep learning-based (AiCE) and iterative (AIDR-3D) image reconstruction algorithms vary according to different brain areas.

## 1. Introduction

Unenhanced brain computed tomography (CT) is a widely used medical imaging technique with a crucial role in the diagnosis, monitoring, and treatment of neurological disorders and injuries [1]. Its ability to provide detailed structural information in a non-invasive and low-cost manner, with quick turnaround times and wide availability, makes it the examination of choice in the emergency setting [2]. However, the acquisition of high-quality CT images is often hindered by various factors, such as beam hardening, streak, and partial volume artifacts observed in the posterior cranial fossa, image noise, and limited variation of Hounsfield units (HU) in subtle pathologic conditions. Moreover, repeated CT examinations are related to cumulative radiation exposure.

Filtered back projection (FBP) is a commonly used reconstruction technique in CT. While FBP has been the go-to method for many years, it does have some limitations that can affect image quality and diagnostic accuracy [3]. For example, FBP does not incorporate advanced noise suppression techniques; as a result, the inherent noise in the raw projection data can be amplified during the reconstruction process, leading to grainy or speckled images. This can obscure subtle anatomical features and reduce overall image quality, particularly when using low-dose CTR protocols. Moreover, FBP lacks sophisticated algorithms for artifact correction; thus, image artifacts can be easily caused by several factors, such as metallic implants, beam hardening, and patient motion.

Iterative reconstruction techniques were introduced to overcome these limitations of FBP. By iteratively refining the reconstructed image using mathematical models and optimization techniques, these algorithms can mitigate the blurring effect caused by the finite size of X-ray beams and improve the visualization of fine anatomical details, achieving higher spatial resolution compared to FBP [4]. Iterative reconstructions incorporate sophisticated noise suppression algorithms, which effectively reduce noise in the reconstructed images. These methods employ statistical models and regularization techniques to differentiate between true anatomical structures and random noise, resulting in cleaner and less grainy images. Improved noise suppression enhances the visibility of subtle structures and increases the signal-to-noise ratio, leading to improved diagnostic confidence, especially with low-dose CT protocols. However, iterative reconstruction techniques still show some limitations, such as longer reconstruction time due to the iterative nature of these algorithms; moreover, when high reconstruction strength levels are combined with low-dose acquisition CT, the resulting images have a waxy and plastic look or a simply unnatural appearance [5].

In recent years, deep learning (DL) has emerged as a transformative technology in medical imaging [6]. DL methods leverage complex neural network architectures to learn intricate patterns and features directly from vast amounts of training data, enabling them to achieve high performances in various imaging-related tasks, one of them indeed being the improvement of image reconstruction [7,8]. Of note, the training process behind DL-based image reconstruction can deal with a fundamental issue of iterative reconstruction, i.e., the loss of algorithm convergence due to the high number of factors that must be balanced during iteration [9].

DL-based reconstructions, with the True Fidelity (GE Healthcare, Chicago, IL, USA) and the Advanced Intelligent Clear-IQ Engine (AiCE, Canon Medical Systems, Otawara, Japan) algorithms, have been extensively investigated in abdominal imaging [8] and have also been the object of pilot studies in unenhanced brain CT for adult and pediatric populations. For example, comparisons of image quality between iterative reconstructions and the True Fidelity algorithm have been reported in adult populations by Kim et al. [10] and Alagic et al. [11] in adult patients, and by Sun et al. [12] and Lee et al. [13] in pediatric patients. In all these studies, DL-based reconstructions significantly reduced image noise and improved image quality, both from a qualitative and quantitative point of view. The AiCE algorithm has been tested in unenhanced brain CT by Oostveen et al. [14]. Their qualitative evaluation found that in four out of five image quality characteristics, DL-based reconstructions outperformed hybrid iterative reconstructions, with significant differences in noise reduction and gray matter–white matter (GM-WM) differentiation. While no statistically significant difference in overall image quality rating was found, DL-based reconstructions outperformed iterative reconstructions in all three analyzed quantitative parameters.

The clinical relevance of image quality improvements granted by DL-based image reconstruction algorithms is particularly high in the emergency setting [15,16]. However, most of the published studies include a heterogeneous case mix; a more homogeneous correspondence with the neuropathological scenario found in the emergency setting has been implemented only in the study by Kim et al. [10], which was, however, solely focused on the True Fidelity algorithm.

Thus, this study aims to explore the application of the AiCE algorithm on a set of consecutive brain CT examinations performed in the emergency setting. In particular, after a preliminary phantom study, the image quality of AiCE reconstructions will be quantitatively compared with that of hybrid iterative reconstructions (AIDR-3D) in terms of image noise values, image noise reduction attributable to the AiCE algorithm, artifact indexes, and contrast-to-noise ratios (CNRs).

## 2. Materials and Methods

This single-center exploratory retrospective study was conducted at the Radiology Department of Fatebenefratelli Hospital (ASST Fatebenefratelli Sacco, Milano, Italy) after approval from the Internal Review Board (protocol code DLBRI, approved on 30th April 2023). All patients provided their informed consent for CT execution and use of their de-identified images.

A preliminary phantom study was conducted by scanning quality control phantoms to assess the basic image quality performances in a well-known and controlled environment. The phantom used for the determination of the spatial resolution was a Catphan 600 unit, whereas a cylindrical water-filled phantom was used for the analysis and quantification of noise on the scans.

Then, for the in-human study, unenhanced brain CT scans of consecutive patients aged > 18 years requested by the Emergency Department of our institution for suspected stroke or after head trauma and performed between 10 April and 12 April 2023, were retrospectively retrieved from the institutional picture archiving and communication system (PACS).

### 2.1. Phantom Study

#### 2.1.1. Spatial Resolution

The Catphan 600 phantom was scanned on a 320-detector row CT scanner (Aquilion One PRISM edition, Canon Medical Systems) on the CTP 591 section with the following exposure parameters: helical acquisition; tube voltage 120 kV; tube current exposure time product 125 mAs; pitch 0.625; collimation 320 × 0.5 mm.

The images were reconstructed in two datasets, the first with the iterative AIDR-3D FC26 kernel and the second with the DL-based AiCE Brain LCD kernel. The following reconstruction parameters were kept unmodified in both datasets: slice thickness 0.5 mm; field of view 22 cm.

On each dataset, five images in the center of the CTP 591 section were extracted. The images were analyzed with IQWorks v0.7.2 (IQWorks Project, University of Edinburgh, Edinburgh, UK) and a point spread function analysis was performed for each image centered on the tungsten wire on CTP 591. A task transfer function (TTF) was determined for each image. The five functions were averaged, and the resulting function was taken as the representative TTF of the dataset.

#### 2.1.2. Noise Analysis

The cylindrical water-filled phantom was then scanned with the following exposure parameters: helical acquisition; tube voltage 120 kV; tube current exposure time product 360 mAs. Again, the images were reconstructed in two datasets, the first with the AIDR-3D FC26 kernel and the second with the AiCE Brain LCD kernel, keeping for both datasets the following reconstruction parameters: slice thickness 0.5 mm; field of view 22 cm; 512 × 512 (rows × columns) matrix.

On each dataset, twenty images in the middle of the phantom were extracted. A 200 mm³ ROI was drawn in the center of each image, and the standard deviation of the ROI was registered as a measure of the amount of noise. Each image was then analyzed with the Qadistri plugin of ImageJ (ImageJ, U.S. National Institutes of Health, Bethesda, MD, USA) to determine the noise power spectrum (NPS). The spectra in the same set were averaged to reduce the statistical fluctuations in the data and determine the NPS of the dataset.

The average frequency of the NPS was calculated and registered as a measure of the noise texture appearance.

### 2.2. In-Human Study

All acquisitions were performed on the same 320-detector row CT scanner (Aquilion One PRISM edition, Canon Medical Systems). All examinations were acquired in helical mode with the following acquisition parameters: tube voltage 120 kVp; tube current 125 mAs; rotation time 1 s; collimation 0.5 × 40 mm; pitch 0.625; field of view 220 mm; 512 × 512 matrix. The effective dose was calculated through the dose length product multiplied by the appropriate conversion coefficient [17].

**Figure 1:**
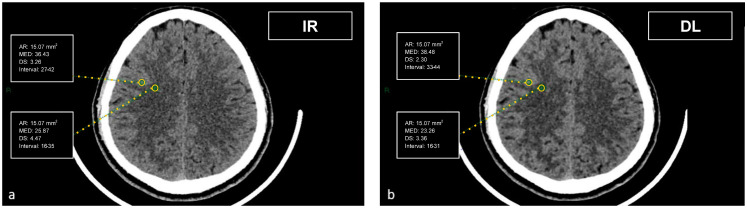
Demonstration of the region of interest placement in the frontal cortex and adjacent WM on iterative reconstructions (**a**) and artificial intelligence-based reconstructions (**b**).

**Figure 2:**
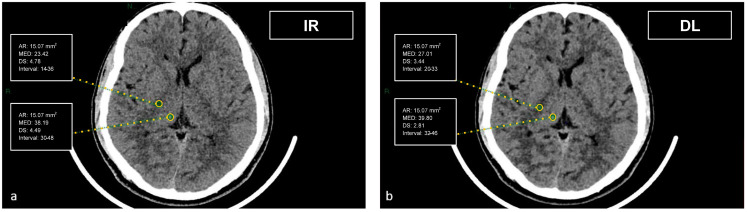
Demonstration of the region of interest placement in the right thalamus and adjacent WM of the internal capsule on iterative reconstructions (**a**) and artificial intelligence-based reconstructions (**b**).

**Figure 3:**
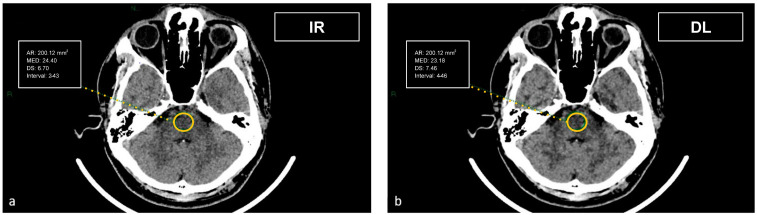
Demonstration of the region of interest placement in the intrapetrous region to assess the image noise in the posterior fossa on iterative reconstructions (**a**) and artificial intelligence-based reconstructions (**b**).

**Figure 4:**
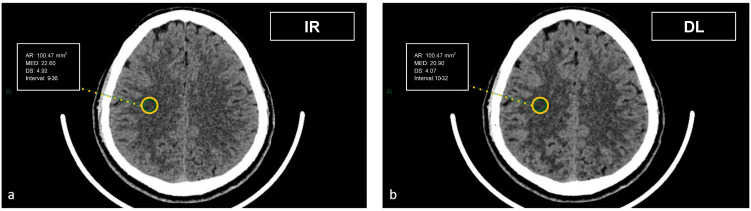
Demonstration of the region of interest placement in the right centrum semiovale on iterative reconstructions (**a**) and artificial intelligence-based reconstructions (**b**) to assess the image noise.

CT datasets were first reconstructed at 0.5 mm with the iterative reconstruction algorithm (AIDR-3D) and then, always at 0.5 mm, with the DL-based reconstruction algorithm (AiCE). Image datasets were visualized on the institutional PACS (Carestream Vue PACS v12.2, Carestream Health, Rochester, NY, USA). To analyze the GM-WM differentiation, four regions of interest (ROI) of 15.07 mm² were delineated: frontal WM and adjacent cortical GM at the level of the centrum semiovale, and thalamic deep GM and WM of the adjacent posterior limb of the internal capsule (Figure 1 and Figure 2). To evaluate artifacts in the posterior cranial fossa, another ROI of 200 mm² was drawn in the inter-petrous region of the posterior fossa at the pons level, where the most noticeable artifacts were seen (Figure 3). All ROIs were delineated by two board-certified neuroradiologists and then reviewed by another board-certified neuroradiologist. We defined CT numbers (HU) of the thalamic deep GM and the frontal cortex as CT attenuation values of GM, whereas image noise was defined as the standard deviation (SD) of attenuation values measured in the deep WM at the centrum semiovale level in a ROI of 100 mm² (Figure 4).

The artifact index was defined as the SD within the ROI of the posterior cranial fossa, which is prone to beam hardening, streak, and/or partial volume artifacts. Therefore, the artifact index may reflect the amount of CT number variations caused by artifacts in addition to the inherent image noise associated with scanner- and patient-related factors.

The CNRs at the centrum semiovale and basal ganglia levels were calculated with the following formula:

$$\frac{Mean\;HU_{GM}-Mean\;HU_{WM}}{\sqrt{Mean\;SD_{HU_{GM}}{}^{2}-Mean\;SD_{HU_{WM}}{}^{2}}}$$

The noise reduction rate of AiCE (%) was calculated as follows:

$$\frac{SD_{AIDR–3D}-SD_{AiCE}}{SD_{AIDR–3D}}\times 100$$

### 2.3. Statistical Analysis

The normality of AIDR-3D and AiCE data distributions obtained from the analysis of human CT images was evaluated with the Shapiro–Wilk test, which confirmed the non-normal distribution of these data. Thus, the non-parametric Wilcoxon test was used for all comparisons between the two sets of measurements, considering their continuous and paired nature. Values of image noise, artifact index, and cortical and thalamic CNRs in the two sets of measurements were expressed as the median and interquartile range (IQR). In order to account for multiple comparisons, the Bonferroni correction was applied considering the four aforementioned tests, with an ensuing *p* < 0.013 threshold for statistical significance. RStudio (with R version 4.2.1, The R Foundation for Statistical Computing, Vienna, Austria) and STATA (version MP 17.1, StataCorp, College Station, TX, USA) were used to perform all analyses.

## 3. Results

### 3.1. Image Analysis in Phantom

In the phantom study, the spatial resolution of the two datasets resulted in being comparable: the TTF of the AIDR-3D dataset showed a 50% frequency of 4.0/cm, whereas the TTF of the AiCE dataset showed a 50% frequency of 3.9/cm.

Conversely, the AIDR-3D reconstruction algorithm resulted in a broader noise pattern: the analysis of the noise amount resulted in an average noise of 6.0 ± 0.2 for the AIDR-3D dataset, whereas for the AiCE dataset the average noise was 4.1 ± 0.2. The NPS analysis showed that the average noise frequency for the AIDR-3D dataset was 2.3/cm, while for the AiCE dataset the average frequency was 1.8/cm.

### 3.2. Image Analysis in Patients

**Figure 5:**
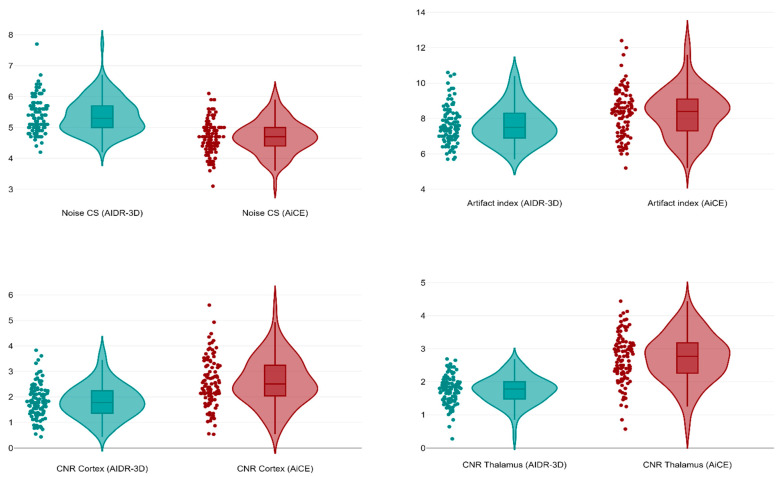
Violin plots comparing image noise in the centrum semiovale, artifact index in the posterior fossa, and CNRs at cortical and thalamic levels between AIDR-3D and AiCE reconstructions.

A total of 100 patients (40 females, overall median age 67 years, IQR 41–80) undergoing unenhanced brain CT for suspected stroke or head trauma were retrieved and included in this study. The mean dose length product was 1028 mGy × cm, whereas the mean effective dose was 2.06 mSv. As shown in Figure 5, in the deep WM of the centrum semiovale the AiCE images had a significantly lower median image noise (4.7, IQR 4.4–5.0) than the AIDR-3D ones (median 5.3 IQR 5.0–5.7, *p* < 0.001), with a median 19.6% noise reduction (IQR 11.7–28.0%, minimum −11.5%, maximum 50.0%). Conversely, AiCE images had a significantly higher median artifact index in the posterior cranial fossa (8.4, IQR 7.3–9.2) compared to AIDR-3D images (median 7.5, IQR 6.9–8.3, *p* < 0.001). However, AiCE images had a significantly higher median CNR both at the cortical level (2.5, IQR 2.0–3.3, *p* < 0.001) and the thalamic level (median 2.8, IQR 2.3–3.2, *p* < 0.001) compared to the median AIDR-3D CNRs of 1.8 (IQR 1.4–2.3, cortical level) and 1.7 (IQR 1.5–1.8, thalamic level).

## 4. Discussion

This study aimed to quantitatively compare the image quality of unenhanced brain CT images reconstructed with a DL-based (AiCE) and a hybrid iterative reconstruction algorithm (AIDR-3D), two types of reconstruction algorithms that are nowadays widely applied in routine clinical practice.

Our study demonstrated that DL-based reconstructions can significantly improve the CNR both at the frontal lobe and basal ganglia levels and provide a low image noise at the supratentorial level, with a mean noise reduction of 19.6%. These findings are consistent with previous studies evaluating CT image reconstruction using DL-based algorithms in the brain [9,10,11,12,13,14] and other anatomical regions [7,8,18,19,20,21]. These results at the supratentorial level confirm the utility of DL-based reconstructions, especially in the emergency setting, where subtle definition of anatomical structures boosts the diagnostic performance of CT in the detection of ischemic and hemorrhagic alterations.

However, we observed that AiCE reconstructions had a higher median artifact index in the posterior cranial fossa compared to AIDR-3D images. The crucial relevance of the posterior cranial fossa in unenhanced brain CT is based on two different aspects: first, because the presence of several delicate structures (such as the brainstem and the cranial nerves) in a small area implies a considerable diagnostic challenge in the emergency setting for the rule-out of hemorrhagic and ischemic events; second, because the posterior cranial fossa is typically affected by artifacts due to the predominant presence of neighboring bony structures [15,22]. This point deserves careful consideration, as the study by Oostveen et al. [14] using the same DL-based algorithm had found a significant reduction of artifacts in the posterior cranial fossa; however, the ROI positioning chosen by Oostveen et al. [14] (in the fourth ventricle) is much less challenging than the placement in the pons implemented by Kim et al. [10] and also adopted in this study, as this positioning is closer to skull base bones. Notably, the latter option is better suited to mirror clinical necessities, as the emergency setting requires the ability to appropriately evaluate the margins of the posterior cranial fossa, which are a particularly problematic area from a clinical point of view. On the one hand, this different ROI positioning in the posterior cranial fossa might, therefore, explain the higher presence of artifacts observed in our study compared to Oostveen et al. [14]; on the other hand, this indirect observation of a worse performance of the AiCE algorithm compared to other studies with the True Fidelity algorithm and the same ROI positioning in the posterior cranial fossa [10,11]—which had found significant reductions of image artifacts—warrants the need for studies comparing different DL-based reconstruction algorithms.

Overall, the results of this study confirm that DL-based reconstructions are able to quantitatively improve image quality in selected areas of the supratentorial gray and white matter, particularly in regions such as the frontal lobe and basal ganglia, which are common sites of pathological processes encountered in the emergency setting. Conversely, the less encouraging results obtained in equally critical but more challenging areas suggest that iterative reconstructions still have an advantage when we intend to compare some quantitative parameters—such as the artifact index—which are nearer to a qualitative assessment and where the long-standing familiarity of radiologists with images from iterative reconstructions still plays a part. However, the capabilities of DL-based reconstruction algorithms, provided that adequate training is performed, should be well-suited to overcome these limitations. Furthermore, the extreme complexity of the cranial anatomical environment implies different necessities according to different areas: the ductility of DL-based techniques could lead to the development of blended models in which, according to the clinical context, optimal reconstruction performance can be achieved through the selection of reconstruction models according to different anatomical regions.

This study has limitations. First, only thin-slice reconstructions (0.5 mm) were evaluated: the impact of DL-based reconstructions on thicker slices is expected to be similar, albeit with less pronounced noise reduction, since thicker slices inherently have less noise regardless of the reconstruction method. Second, image noise was only measured in terms of standard deviations; this does not account for different noise textures associated with each reconstruction algorithm, which play a role in the perception of “natural” image appearance. Third, our evaluation was limited to adult patients, even though the benefits of DL-based reconstruction algorithms are foreseen to be even higher in pediatric patients, where the application of low radiation dose protocols favors the emergence of imaging artifacts.

In conclusion, data from this study show how the performance of DL-based (AiCE) and iterative (AIDR-3D) image reconstruction algorithms for unenhanced brain CT varies according to different brain areas. Wherever anatomical complexity poses a particular challenge to CT image reconstruction in the emergency setting, the combined opportunistic use of hybrid iterative and DL-based appears as a viable option to achieve optimal image quality until further development of DL-based models leads to high-level image reconstruction in all regions of the brain.

## Data Availability

All analyzed data are included in this paper.
